# Neighbourhood Built Environment Influences on Physical Activity among Adults: A Systematized Review of Qualitative Evidence

**DOI:** 10.3390/ijerph15050897

**Published:** 2018-05-02

**Authors:** Grazia Salvo, Bonnie M. Lashewicz, Patricia K. Doyle-Baker, Gavin R. McCormack

**Affiliations:** 1 Department of Community Health Sciences, Cumming School of Medicine, University of Calgary, Calgary, AB T2N 4Z6 Canada; bmlashew@ucalgary.ca (B.M.L.); gavin.mccormack@ucalgary.ca (G.R.M.); 2 Faculty of Kinesiology, University of Calgary, Calgary, AB T2N 1N4, Canada; pdoyleba@ucalgary.ca; 3 Faculty of Environmental Design, University of Calgary, Calgary, AB T2N 1N4, Canada

**Keywords:** physical activity, built environment, qualitative, neighbourhood, walkability

## Abstract

Qualitative studies can provide important information about how and why the built environment impacts physical activity decision-making—information that is important for informing local urban policies. We undertook a systematized literature review to synthesize findings from qualitative studies exploring how the built environment influences physical activity in adults. Our review included 36 peer-reviewed qualitative studies published from 1998 onwards. Our findings complemented existing quantitative evidence and provided additional insight into how functional, aesthetic, destination, and safety built characteristics influence physical activity decision-making. Sociodemographic characteristics (age, sex, ethnicity, and socioeconomic status) also impacted the BE’s influence on physical activity. Our review findings reinforce the need for synergy between transportation planning, urban design, landscape architecture, road engineering, parks and recreation, bylaw enforcement, and public health to be involved in creating neighbourhood environments that support physical activity. Our findings support a need for local neighbourhood citizens and associations with representation from individuals and groups with different sociodemographic backgrounds to have input into neighbourhood environment planning process.

## 1. Introduction

Physical activity is an important determinant of health, wellbeing, and disease prevention. Regular moderate-to-vigorous intensity physical activity (MVPA) can improve metabolic, cardiovascular, oncological, musculoskeletal, and psychological function and can reduce the risk of chronic conditions including cardiovascular disease, some cancers, type II diabetes, hypertension, stroke, depression, and overweight and obesity as stated previously [1]. However, despite the numerous health benefits of physical activity (PA), many adults in high-income countries such as Canada, U.S., and Australia do not participate in levels of PA sufficient to accrue optimal health benefits. As part of their Global Strategy on Diet, Physical Activity, and Health, the World Health Organization recommends that adults accumulate at least 150 min of MVPA per week undertaken for leisure, transportation, work, play, sports, and exercise [2]. 

The determinants of PA are multi-faceted and complex. Over the past few decades there has been increasing research and political interest in the role of the built environment (BE) in supporting PA [3]. The BE is human-made and consists of the distribution of buildings and designed spaces that support activities, the services and infrastructure of the transportation system (including roads, sidewalk, bike paths), and urban design [4]. Findings from quantitative studies suggest that neighbourhood built characteristics including street or pedestrian connectivity, the mix of destinations and land uses, population and residential density are consistently associated with PA and in particular walking [5,6,7] since dense neighbourhoods that are connected and offer nearby destinations may increase transportation walking. While less consistent, there is also quantitative evidence suggesting that neighbourhood park space, safety and aesthetics, features associated predominantly with leisure walking, may support PA [4,6,8].

There have been recent calls for more evidence on the BE and PA that is policy relevant and that can be easily implemented in urban planning and design [3]. Quantitative evidence shows that there are relationships between the BE and PA. This evidence is important to inform urban planning policy and practice however, quantitative studies provide limited insight and understanding about the day-to-day lived experiences and the interactions adults encounter with potential BE constraints and enablers when they attempt to be physically active in their neighbourhood. Exploration of people’s experiences with the BE in relation to their PA can provide new and different knowledge, which might be of importance for local urban planning decision-making. For example, a review of qualitative studies of park environments and PA [9] supported findings from the quantitative evidence [10,11]. The review findings also contributed novel insights suggesting that the influence of park availability and proximity on PA while important, is often constrained by perceived and actual personal safety concerns and that the delineation between built versus social environment determinants of PA is not always clear. 

Qualitative research can therefore provide important contextual insights about the determinants of PA [12,13], including the influence of supportive and constraining neighbourhood built characteristics that could inform local urban planning and policy. Given that much of the quantitative evidence on BEs and PA is cross-sectional, thus limiting causal inferences, qualitative evidence has the potential to illuminate the plausibility of this relationship including under what individual-level and social conditions the BE enables or inhibits PA [13]. Further, built characteristics found to be associated with PA, such as interesting destinations and aesthetics, can vary according to people’s perceptions, personal experiences, and attitudes [14], and may not be fully discernable when these perceptions are statistically summarized as is the case in quantitative studies. Qualitative studies explore and describe experiences and lead to insight about how adults perceive and interact with their neighbourhood environments in their attempts to be physically active. Therefore, the purpose of our study was to undertake a systematized literature review to synthesize qualitative research findings on how the neighbourhood BE influences PA in adult populations.

## 2. Materials and Methods 

### 2.1. Overview

Systematized literature reviews have been undertaken previously to explore the relations between built and social environments and health outcomes [15] including PA [16]. Our study included a systematized literature review, synthesizing qualitative evidence. Like systematic reviews, the article search and selection, data extraction, and results synthesis for systematized reviews are determined a priori, fully documented, and systematic. However, a systematized review is often distinguished from a systematic review in that the former may not include a formal assessment of study quality, remove or weight study findings based on methodological quality, nor pool results to undertake meta-analysis [17]. Qualitative evidence does not lend well to the tools and approaches designed primarily to appraise and summarize the internal validity of quantitative evidence [18]. Despite not applying a formal qualitative appraisal, in our review we synthesized and compared the different qualitative methodologies to inform our valuation of credibility and trustworthiness of findings within this literature. 

### 2.2. Search Strategy

In April 2016, we searched for English-language peer-reviewed qualitative studies, with no publication date restrictions, that had reported associations between the BE and PA. Given the interdisciplinary nature of this research topic, we searched for relevant articles within health (PubMed, MedLine, PsycInfo, and SPORTDiscus), leisure (Leisure Tourism Abstracts), urban planning (Urban Studies: Environmental Complete) and transportation (Transport Research Information Services (TRIS)) databases. Within article title and abstracts, we searched for a combination of key terms (and their variant spellings) related to the BE (built environment, spatial, neighbourhood, physical environment, streetscape, urban form, urban planning, walkability, pedestrian-friendly, geographic information systems, parks, and greenspace) [6], PA (physical activity, exercise, inactivity, walk, bicycling, cycle, stroll, run, jog, leisure-time, sport, recreation, active transportation, and pedestrian) [6], and qualitative research (qualitative, focus group, interview, ethnographic, ethnography, case study, and anthropology) [9]. 

### 2.3. Study Selection

Following the database search, *n* = 8237 articles remained after the removal of duplicates. We screened all article titles and abstracts for relevance, and removed non-primary studies (e.g., literature reviews, methodological studies) and article types irrelevant to our purpose (i.e., commentaries, editorials, conference proceedings). Relevance of titles and abstracts led to the selection of 71 articles that underwent full-text review. Following full-text review, articles were included if they reported qualitative findings for an association between the neighbourhood BE and PA (i.e., included either participant descriptions or interviewer interpretation of participant descriptions of the association). Eligible articles must have specifically reported on a primary study that: (1) included at least one qualitative data collection method (i.e., unstructured or semi-structured interviews, focus groups, photovoice methods, qualitative survey); (2) discussed or reported on participants’ experiences of neighbourhood BE barriers and facilitators to PA, and; (3) included an adult sample. Studies that did not include adults or included quantitative findings only were excluded from the review. A total of 36 articles met the inclusion criteria. 

### 2.4. Data Extraction

For each included article, we extracted and reported information regarding data collection method (e.g., interview, focus group), sample characteristics (e.g., sex, age, rural vs. urban, ethnicity, socioeconomic status), analytic approach (e.g., thematic analysis, grounded theory) and findings about the BE’s supporting or restricting role in relation to PA. We used an existing conceptual framework developed by Pikora et al. [19] to guide our initial extraction and reporting of the BE and PA findings. This framework was developed using published evidence and policy literature, interviews with experts and a Delphi study [19]. The framework posits the relations between specific neighbourhood built characteristics and walking in terms of four key features: *functionality*, *safety*, *aesthetics*, and *destinations* [19]. Functional features include direct routes, intersection design, path design and maintenance, traffic control and vehicle parking. Safety features include surveillance, crossing aids and lighting. Aesthetics features include cleanliness, interesting sights, maintenance, greenery, architecture and pollution. Destination features include proximity, accesses, and availability of local facilities, parks, shops, parking facilities, public transit and other destinations. This framework has been used in previous studies investigating the associations between the BE and PA [12,20,21].

## 3. Results

### 3.1. Summary of Study Methods

#### 3.1.1. Study Characteristics and Sample Designs

The 36 studies included in this review were published between 1998 and 2015. Twelve were undertaken in the USA [14,22,23,24,25,26,27,28,29,30,31,32], six in Canada [13,33,34,35,36,37], two in the UK [38,39], eight in Australia [12,40,41,42,43,44,45,46], two in New-Zealand [47,48], and one each in Ireland [49], Brazil [50], Sweden [51], Belgium [52] and Iceland [53], with one study having recruited from both Canada and the USA [54] (Appendix A). Sample sizes varied from eight to 396 participants with four studies not specifying a sample size. Thirty-four studies used purposive sampling frameworks to recruit participants based on: gender (*n* = 16), ethnicity (*n* = 7), socio-economic status (*n* = 9) and/or age with 13 studies focusing on adults older than at least 50 years. Studies that sampled based on ethnicity did so from African American [22,27,29,31], Hispanic/Latino [22,23,31], and American Indians populations [31]. Three studies specifically sampled adults from rural areas [12,34,37].

#### 3.1.2. Data Collection and Analytic Approaches

Focus groups [13,14,26,28,29,31,32,36,38,40,43,47,49,50,51,54] and individual face-to-face interviews [12,22,25,30,39,40,41,42,44,45,46,48,53] were the most common qualitative data collection approach (Appendix A). Five studies included photovoice methods [27,33,34,37,54] in addition to focus groups or interviews. The photovoice method elicits rich data through allowing participants to take photos of their surroundings, then using the photos to tell the stories behind them to the researcher. Two of these studies included walk-along interviews [24,52]. One study used a qualitative questionnaire, which included open-ended questions capturing participants reasons for enjoying PA [35]. One mixed methods study presented qualitative data from semi-structured interviews only [39]. 

Of the analytical approaches, thematic analysis and content analysis were used most frequently. Content and thematic analysis were used somewhat interchangeably, although qualitative methodologists tend to distinguish content analysis as focused on what language is used by participants and with what frequency, while thematic analysis tends to emphasize interpreting participant language in context [55]. Five studies [26,37,44,49,51] identified grounded theory as their analytical approach and used techniques such as constant comparison however, they did not necessarily specify the ways in which their approach led to the theory building expected when employing grounded theory approaches [56]. A phenomenological approach was used in one study [53] as guiding researchers to focus on the essence and structure of participants’ subjective experiences of PA and the BE [56] (Appendix A). 

### 3.2. Relationships between the BE and PA

**Figure 1:**
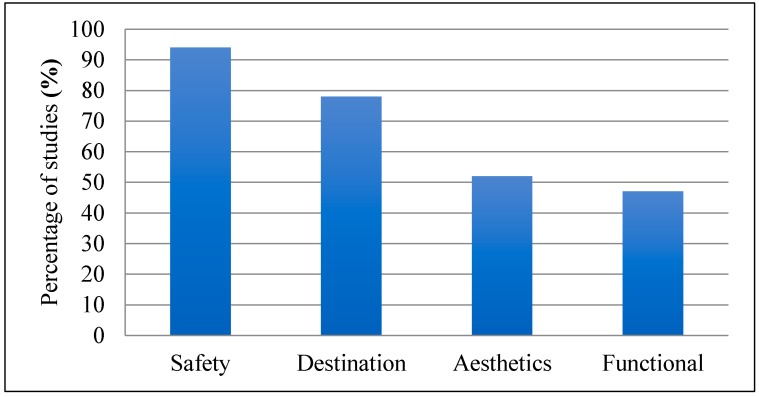
Percent of included studies (*n* = 36) categorized by reported key built environment features.

Based on the categorization of the BE features using Pikora et al.’s [19] framework, most studies included in our review reported on safety, followed by destination, aesthetics and functional features as enabling or limiting physical activity (Figure 1). A summary of findings related specifically to the BE features and PA extracted from the reviewed studies is presented in Appendix B.

#### 3.2.1. Functional Features

● Paths and access to amenities supporting PA and mobility

In several studies participants reported access to sidewalks, paths and walkways as key characteristics that support their walking [27,34,39,54]. In high traffic areas, the presence of pedestrian bridges over large roads helped make walking feasible [24]. In contrast, sidewalks that suddenly ended and had poorly maintained surfaces that were uneven or slippery due to cracks, puddles or ice were barriers to walking [27,39,52,54]. In particular, for older adults where the fear of falling was a barrier to PA, sidewalk cracks, stairs and hills posed challenges [23,37,39,52,53]. For older adults, access to less steep sidewalk ramps were found to be helpful for enabling walking [37,39,53]. As one older woman noted in relation to access to less steep ramps: “At least here you can walk without falling or spraining your ankle, this is all flat” [52]. 

Further, older adults had a special appreciation for amenities such as benches, drinking fountains, public washrooms, railings for stairs, shaded areas and ramp access [27,33,37,39,44,54]. Without these amenities, some pathways and public open spaces were perceived as unsupportive of PA, as one elderly woman expressed: “Of course you want shade in the park. A couple of senior citizens out for a stroll, they want to be able to sit when they get pooped, there is nowhere for them to sit” [33]. Weather conditions such as extreme heat and slippery or snowy winter conditions also posed a falling risk for older adults [23,27,30,39,40,52,53]. Whereas removal of ice and snow on pathways helped older adults to remain active in the winter months [27].

● Path design and connectivity supporting active transportation modes

Several functional features were reported as supporting cycling. For instance, having different types of pathways connect (i.e., connectivity) was considered important for bicycle commuting in an Edmonton (Alberta, Canada) study where some participants reported driving by car to the bike path because of lack of bike path connectivity: “I mean the river valley is beautiful but there’s no way to get there on a bike, I mean there is but you have to drive there with your bike” [13]. Participants also mentioned how separated bike lanes, walking paths and motorized vehicle lanes improve ease of getting around [30,34]. For example: “Now we have wonderful biking facilities and path[s] [separated from motorized traffic] I walk with a friend in the morning … it’s a 2-mile stretch” [30].

#### 3.2.2. Safety Features

● Crime and sense of trust in the community

Participants raised two main safety concerns in relation to the BE: (1) safety from crime, and (2) safety from traffic. If residents perceived the crime rate in neighbourhoods as being high, they were not inclined to walk or participate in physical activities in local public areas such as parks [22,23,27,29,31,32,39,41,43,45,49,52]. In some cases, participants were afraid to leave their homes because of the presence of gangs and drug dealing and this deterred neighbourhood PA [26,31,43,45,49,57]. For example “You’re insane to be outside. You could get shot, robbed, beat up” [26]. Notably, safety from crime was of greater concern among women compared with men [51]. Environments that provide safe spaces from crime were mentioned as supportive places to undertake PA especially among women [22,31,32,43,45,51]. 

Perceived or real lack of safety discouraged participants from being physically active outdoors especially in low-socioeconomic status neighbourhoods [14,26,31,45,50]. Even in these low-socioeconomic status neighbourhoods where facilities and destinations were close or within walking distance to home, the fear of crime was a barrier to PA—as a woman from a low-socioeconomic status neighbourhood noted: “It’s like you’re scared to live here. At a certain time at night ... uh, well everything’s close by there, but the truth is, you don’t feel comfortable living. […] and well that’s what worries us...because sometimes we can’t go out or you don’t feel comfortable going out.” [14]. Non-violent crime and evidence of incivilities including loitering [14], explicit sexual behaviours [14], vandalism [54], lack of cleanliness/littering [26,39,49], cars illegally parked on sidewalks [39], and drug paraphernalia [26,54] also negatively influenced participants perception of safety and subsequently, their neighbourhood PA patterns: “Some problems in the area include poverty, drug and criminal activities and poor housing, etc. Some people do not feel safe or willing to walk through area to get to river” [54]. 

Increased police presence helped some to feel safer in high crime areas [14,26,27,39]: for example “More police presence might enhance use as the parks would be safer” [14]. In contrast, minority groups living in low-income neighbourhoods in the U.S. experienced racial profiling by law enforcement authorities dissuading them from visiting and being physically active in neighbourhood spaces such as parks and recreation centers [23,26]. One resident living in a minority neighbourhood described the racial profiling: “The police intervene unnecessarily when a group of teens hang out at the Rec[reation] Center and profiling makes them think they are a gang” [26]. This contrast may suggest that some neighbourhood built characteristics impact the PA levels of different populations in different ways.

Sense of community, facilitated by the BE, and knowing one’s neighbors contributed to feelings of safety. Residents in low-socioeconomic status areas used social spaces and amenities such as courtyards, picnic tables and BBQs to develop social ties with others in the community [24,44,46]. As Walker and Hiller (2007) describe: “For one woman, being ‘known’ at her local shops contributed to her sense of safety within the area” [44]. An African American woman described her experience in a courtyard as: “If you’re out in a courtyard area, then you see people coming and going, being outside. So, you get to know them. You may not speak the same language, but you know, you do say ‘hi’ to each other. They’ll ask me how I’m doing. I’ll ask them how they’re doing. So, it’s more like a family” [24]. Creating neighbourhood spaces that facilitate social interaction and sense of community may counter the fear of crime and encourage adults to engage in neighbourhood PA.

● Lighting and fear of darkness

Participants preferred being physically active during the day or in well-lit environments and avoided darkness and isolated areas with poor visibility [32,39,42,43,49,54]. In particular, women felt uncomfortable undertaking PA outdoors at night and preferred well-lit areas [31,32,38,43], for example: “So, I sort of think, it’s a Friday evening, do I really want to go for a jog around the park, when there’s going to be groups of lads drinking? And I end up not going” [38]. The perceived need for more lighting seemed to be more important in some low-socioeconomic status neighbourhoods where violent crime and gangs were present [32,45]. Notably, safety concerns for women dissipate as women become more familiar with the neighbourhood and where well-lit and or well populated areas exist [38]. 

● Traffic hazards related to different user type conflict

Traffic hazards deterred transportation walking and cycling [13,30,31,37,43,48,49]. Traffic was a barrier to transportation cycling as expressed by one woman “Too much traffic to bike to town […] it is just scary” [34]. Notably, cyclists themselves were identified as being a hazard for pedestrians: “Most cyclists ride like they’re on a highway. Older persons are frightened or have to step aside” [52]. Separating pedestrians and cyclists from motorized traffic was reported as a means of countering traffic-related safety concerns [30]. Ambiguity regarding right of way between different modes of transportation including motor vehicles, bicycles and pedestrians influenced older adults walking behaviours [36].

Among traffic issues, speeding cars and careless drivers were identified as hazards for walking and cycling [48,52,54]. Older adults felt especially unsafe around traffic and speeding cars [23,30,37,39,48,52,54]. Lack of pedestrian crossings was a barrier to PA [54] and time allowances of crosswalks was considered not long enough [23,33,37]. As one elderly woman in Canada noted: “The major roads, they don’t give sufficient time for you to cross […] doesn’t give you enough time for a person who is elderly, who is immobile to cross, there isn’t a sufficient island for the person to safely stand there” [33].

By comparison, in rural areas, traffic density was less of a problem when compared to concerns related to the presence of large vehicles and winding roads: “But the road … I walked it once and I was terrified. Because it’s sort of a windy road. It’s narrow and you get log trucks” [12]. Barriers to PA in rural areas differed to those found in urban areas.

#### 3.2.3. Aesthetic Features

● Desire to be active in beautiful environments

People were motivated to be active within public environments that were aesthetically pleasing and beautiful [27,39,47]. Contact with greenery whether in the bush, park, garden or courtyard, as opposed to streets, was valued and for many participants, seemed to confer feelings of peace, well-being and restoration [27,34,37,47,48,51,52]. One man described the green spaces in his neighbourhood as important: “That is one of the most important values with this living environment. There are green spaces. […]; These spaces give opportunity to experience the closeness of vegetation and greenness” [51]. Moreover, water elements such as beaches [47], rivers [13] and waterfalls [37] were noted as motivators for PA in public areas. Furthermore, participants expressed preferring to take scenic routes in some cases even if these take more time [27,30,32]. Human-made elements such as architecture and historical monuments were also mentioned for their power to give places meaning and beauty [27,34]. Feelings propelled by aesthetic elements encouraged participants to engage in physical activities such as running or walking in their surroundings as described by a man living in a New Zealand suburb: “No, I love running in the bush and things, I think it’s great, as opposed to running around the streets. I mean I like the character houses, I can do that, but I would much prefer to be in the bush and round the mountain bike parks and places” [47]. On the flipside, unaesthetic areas containing trash and vacant un-kept lots were considered unsupportive to outdoor physical activities as were areas exposed to noise [39,48,52] and air pollution [34,48].

#### 3.2.4. Destination Features

● Availability of, and access to, local destinations and active transportation

Participants reported increased willingness to visit recreation PA destinations such as parks and facilities when these destinations were conveniently located near home [28] and when visiting these destinations did not entail using a car, as Belon et al. highlighted: “several participants who had automobiles reported that they preferred not to drive to distant recreation areas or facilities. Thus, shorter walking distances between home and these areas could encourage PA” [34]. Grocery stores [23,24,39,53,54] and post-offices [39] were considered important facilitators of active transportation. Notably, participants living in a car-friendly city that encouraged car use to run errands, still appreciated having recreational infrastructure such as dog parks and soccer fields within walking distance [13]. 

Proximity to destinations within walking distance became especially important for older adults in cases where they chose, or were forced, to relinquish their motor vehicle operator’s license. Considering the possibility of not being able to drive in the future, one elderly woman describes the importance of proximity to destinations as follows: “[The neighbourhood is] very close to all facilities this is what attracted me. The closeness of shops. Everything else. The bus is only at the corner and the doctor and a dentist and a chemist and a post office and everything, supermarket, everything within a five minute walk” [44]. Older adults valued proximity to shops/markets, post office, food stores, restaurants, libraries, churches, historic monuments, community gardens and parks/green spaces [27,30,33,39,53,54]. Proximity to recreational facilities within the neighbourhood was also considered important and facilities identified include sports fields [13,34], playgrounds [47], green space/parks [24,26,39,54], courtyards with picnic tables [24], festivals [54], community gardens [26], and historical destinations [27]. Access to public transit was mentioned as a factor in supporting walking for transportation and accessing specific PA facilities [34,39,52,54], for example: “I find just even having that [train] makes me get out and be more physical than if I had a car […] I like that I have a little bit of a walk to get to the train” [34].

The lack of nearby PA facilities (i.e., gyms, sports facilities, pools) was mentioned as a barrier to neighbourhood PA [25,26,39]. Further, poor quality of, and lack of participant knowledge about exercise machines in such facilities [41], as well as lack of participant knowledge about availability of recreational facilities in the neighbourhood [26], along with limited operating hours of recreational facilities such as pools, were potential barriers to PA [34]. Not only is the structure and proximity of recreational destinations important, but the operations of recreational facilities (available support for using equipment or facilities, hours of operation, user fees of costs) have the potential to enable or discourage PA. 

● Social interactions at destinations supporting PA behavior

Destinations that are close to home and offer socializing opportunities appeared to motivate participants to be physically active in their neighbourhoods. Some destinations, such as parks and community gardens, were valued as a safe space for PA and creating social ties [24,54]. Inclusive environments where neighbours were friendly and inclined to greet one another were preferred destinations for walking [27,51]: “I walk around the track.... It is really nice, and you see people are running or jogging” [27]. Older adults also considered local destinations to be important for PA and socializing [23,53]. In addition environments affording opportunities to see familiar faces [27,30,44,52], those affording contact with nature and wildlife were important, as noted by a 93-year old: “I prefer to sit outside, its not so lonely being outside in the open. You can hear the birds, not so lonely as always being by yourself inside” [44]. Retirement offered older adults more time to spend engaging in the neighbourhood as a means to connect with others while staying active [36]. Older adults were found willing to actively travel up to 45 min to get to their destinations if the proper infrastructure was in place to engage in pleasurable and purposeful activities: “Now I have enough time, so I can get on my bike and go to the library, and bike to church, or bike wherever I want to.” [30]. Even if they have the time, however, if barriers are present, older adults are less likely to engage in an outdoor PA: “[Even with time] the absence of sidewalks, high traffic … I won’t walk, why would I?” [30].

Destinations were also important for some ethnic minority groups where, for example, cultural forms of PA such as traditional dances, were a preferred means of PA and socialization [31,32]. As noted by an American Indian woman: “I go to a pow-wow with my grandchildren and dance!” [32].

## 4. Discussion

Our review of qualitative studies, similar to previous reviews of quantitative [58,59] and qualitative evidence [9,60], confirmed the importance of the BE for influencing different types of PA (transportation walking, recreational walking, bicycling, running, sports, and other outdoor activities) among adults. For example, street connectivity and nearby destinations were consistently acknowledged in the qualitative studies reviewed as important for supporting transportation-related PA. Functional features that increase street or pedestrian connectivity, that create PA opportunities for all physical abilities and age groups, and that support different transportation modes were important for supporting PA. Safety features such as lighting, the creation of safe public areas for socializing, and infrastructure that separate pedestrians, cyclists and motorized traffic were found to positively impact PA. Aesthetic features including natural elements (e.g., vegetation, waterfalls, beaches), greenery, and the presence of interesting destinations were important for motivating people to be physically active, increasing the time people spend outside, and for providing restorative benefits to people while they walk for leisure, bicycle, and participate in other outdoor activity. 

Beyond the BE and PA relationships posited by Pikora et al.’s [19] conceptual framework, our review findings illuminated the lived experience shaping associations between the BE and PA. Specifically, age and other sociodemographic characteristics contributed to perceived BE enablers and barriers of PA, and in some cases the BE even had a differential effect on PA of individuals depending on their sociodemographic characteristics. For instance, neighbourhood built characteristics related to police surveillance made some people feel safe and others feel racially profiled and impacted the PA levels of different populations in different ways via potentially different means (e.g., informed or modified by cultural and social norms and stereotypes). Moreover, individuals living in rural areas had unique challenges that were not experienced in urban areas such as wide roads and the presence of trucks. Thus, the BE design strategies for improving the PA supportiveness of urban areas likely differ to the strategies that might improve PA in rural areas. Older adult perspectives on BE enablers and barriers on PA were consistent with findings from a previous review of qualitative evidence, which found pedestrian infrastructure, safety, aesthetics, and access to nearby destinations, rest areas with benches, and washrooms to influence PA [60]. We found that safety and functionality features and destinations were important for supporting recreational and transportation PA among older adults. 

Fear of falling was a major concern among older adults. Environments, including slippery floors, poor lighting and uneven surfaces, are a major risk factor for falls in older adults [61]. Some older adults restrict their PA, as well as activities of daily living, because of their fear of falling [62], which in turn can increase the risk of falls because of the decline in muscle strength and proprioception that accompanies decreased PA during the aging process [63]. Because of conditions such as chronic diseases and limited mobility, older adults sometimes reported pathways and sidewalks with uneven slippery surfaces, and no amenities such as benches, washrooms and railings, heightened their fear of falling and subsequently limited their PA. Our review findings however, are limited in that they do not report how differences in culture and health status can affect the importance of these factors in older adults. Our review findings also highlight the importance of creating neighbourhood BEs that allow ‘aging in place’. For instance, destinations within walking distance to home that support PA were used more often by older adults. Signage targeted towards road users indicating that elderly people are in the vicinity might help older adults feel safer while crossing roads [23]. As the world’s population is rapidly aging, the World Health Organization is emphasizing the importance of engaging cities to promote active aging in place [64]. Shorter walking distances to destinations and amenities in supporting active transportation became more important with advancing age. This is important because active transportation is associated with improved overall fitness and health (reduced BMI, hypertension, waist circumference, triglycerides, stress) [65,66,67,68].

Findings from our qualitative review highlighted consistent evidence regarding the importance of the social environment, and notably built characteristics that encourage or enable social interactions, in supporting PA. The importance of the social environment for supporting PA has also been found in quantitative studies [5]. The presence of social spaces where neighbours could meet one another provided individuals with a sense of safety in neighbourhoods that were perceived to be unsafe by allowing individuals to become familiar or positively interact with one another and by providing passive surveillance. Building trust in the community through social events and social spaces could improve PA by making residents feel safer. Other factors that may contribute to perceived sense of safety include BE characteristics (lighting and maintenance), individual characteristics (gender and age), passive surveillance (likelihood that neighbours are watching), and the time of day [8]. The multifaceted evidence from our review reveal that social spaces also motivate individuals to be active when these spaces offer opportunities to see friendly faces. In addition to providing a sense of community, knowing that others (even if not known by name) in the community may be watching them could provide sense of safety. Previous quantitative studies report inconsistent findings regarding associations between sense of community and PA, however these studies were limited as sense of community is difficult to measure [8]. Environmental characteristics that have been associated with heightened sense of community include low residential density, mixed land use and high walkability [69]. However, objective measures of low residential density are typically correlated with low walkability, thus our study illuminates the complexity of sense of community, PA and neighbourhood built design [5,6,7]. Our findings show for example that neighbourhood cultural activities such as pow-wows could also help residents develop a sense of community. Thus, in neighbourhoods with a predominant ethnic group, customizing the PA opportunities to cultural needs and creating culturally appropriate opportunities for socializing such as traditional dances could contribute to the sense of community, perceived safety and increased PA.

Our review findings reinforce the need for synergy between transportation planning, urban design, landscape architecture, road engineering, parks and recreation, bylaw enforcement, and public health to be involved in creating neighbourhood environments that support PA [3]. Our findings, also suggest that there is a need for neighbourhood citizens and associations with representation from local individuals and groups with different sociodemographic backgrounds to have input into neighbourhood environment planning process. The process of engagement and actual engagement of local residents can often impact if and what BE modifications occur in neighbourhoods [70]. Our qualitative findings support the use of community planning protocols that incorporate both qualitative assessment of the built environment through community engagement alongside quantitative assessment through community audits for planning physical activity supportive communities. Similar to previous reviews [9,60], our findings suggest that neighbourhood physical infrastructure that supports PA is important, but not a sufficient enabler for PA and that the sociodemographic profile of the neighbourhood as well as other social environmental, cultural, and historical factors need to be considered when promoting PA. PA interventions informed by the socioecological framework [71] that target individual, social environmental, physical environment, and policy and regulatory determinants are more likely to encourage behaviour change. Previous research shows that combining BE changes with other health promotion and behaviour change strategies can be successful in increasing PA in adults [72]. For example, a community strategy to promote walking that involved health marketing, health promotion strategies from health care providers and environmental strategies, such as installment of signage and pathways repairs, lead to modest increases in PA in women [72].

Our findings are impacted by the methodological rigor and limitations of the individual qualitative studies included in our review. Although no studies were excluded for methodological reasons, transparency of sources, analysis, reflexivity and rich data [73] were not always described in the studies included in our review. Moreover, we included both participant quotations and authors’ interpretations in our analysis. Thus, both the points of view of the authors of the original studies and the qualitative findings presented in these studies influenced our synthesis and interpretations of findings. Despite incorporating qualitative evidence only, our review is not impervious to publication bias—studies with uninteresting, or conventional findings in relation to the BE and PA may be underrepresented in our review because they have not been published in the peer-reviewed literature. It is possible that by including published peer review qualitative studies only in our review; the association between the BE and PA could be overemphasized. 

Nevertheless, our general findings reflect those reported in other quantitative and qualitative reviews, that is, the neighbourhood BE-PA association exists. The fact that individuals report specific BE characteristics as barriers and enablers to their physical activity and can often describe in detail their positive and negative lived-experiences in this regard, could also imply that for these individuals the relations between the BE and PA are not only plausible, but in some cases, causal. In addition, although the studies included originated from several areas in the world, the results were consistent in that safety, aesthetics, destinations and functional features acted as barriers and enablers of PA.

## 5. Conclusions

Our review explores for lived experiences in adults’ current neighbourhood environment in relation to PA decision-making and behaviour. Notably, no studies included in our review captured participant’s lived experiences in relation to changes in neighbourhood environment and PA change over time (e.g., as part of residential relocation studies or natural experiments). There have been calls for more natural experiment research investigating the relations between the BE and PA to better evaluate temporal causal pathways [6,13]. While these calls seem to emphasize quantitative methods, there similarly needs to be more natural experiments that incorporate qualitative methods. Natural experiments are recommended to understand the impact of small and large-scale urban planning interventions on health; however, such experiments may be vulnerable to bias. Combining methods is one recommended way of addressing some of this bias and contributing to the plausibility of causal inferences [74]. Mixed method study designs within natural experiments, such as those that have been used in recent park- and transportation-related studies [75,76,77,78] will provide a fuller understanding regarding the plausibility of the causal relations between the BE and PA. 

## Appendix A

**Table A1:** Sample design, data collection approach, and analytical approach methods for studies included in the review (*n* = 36).

Author, Year of Publication, Reference	Study Location	Sample Design (n)	Sample n for Qualitative Data Collection	Qualitative Data Collection Approach	Analytical Approach
Cleland et al. (2015), [12]	Australia	Rural dwelling adults 18–55 years	49	Semi-structured interviews	Thematic analysis
Mitra et al. (2015), [33]	Canada	Elderly adults > 65 years	14	Photovoice and interviews	Thematic analysis
Mama et al. (2015), [22]	USA	African American/Hispanic middle-aged women (age M = 43.9 ± 7.3 years)	8	In-depth interviews	Thematic analysis
Ivory et al. (2015), [47]	New Zealand	Adults 18–65 years from varying neighborhood walkability and deprivation	Not stated	Focus groups	Thematic analysis
Marquez et al. (2014), [23]	USA	Older Latino adults > 50 years	20	Exploratory focus groups	Thematic analysis
Belon et al. (2014), [34]	Canada	Adults > 16years from four communities both rural and urban	35	Photovoice and semi-structured interviews	Thematic analysis
Walton E. (2014), [24]	USA	Adults 20–79 years of low income neighborhoods	27	Walk-along interviews and focus groups	Thematic analysis
Shuval (2013), [25]	USA	Low income ethnic minority urban adults 30–54 years	25	Qualitative interviews	Thematic analysis
Bellows-Riecken (2013), [35]	Canada	Undergraduate students, mean age 22.26 years	126	Qualitative written questionnaire	Thematic analysis
Eriksson et al. (2013), [51]	Sweden	Adults 18–84 years	28	Focus Groups	Grounded Theory
Kilgour et al. (2013), [38]	UK	Women 18–62 years	10	Group and individual interviews	Not stated
Bjornsdottir et al. (2012), [53]	Iceland	Women > 70 years living in retirement community	10	Interviews in home or retirement centre	Phenomenology
Lord et al. (2012), [40]	Australia	Men > 45 years	65	Focus group and semi-structured interviews	Thematic analysis
Mahmood et al. (2012), [54]	Canada/USA	Older adults > 65 years in Vancouver and Greater Portland	66	Photovoice and group discussion.	Thematic analysis
Stathi et al. (2012), [39]	UK	Adults > 70 years	25	Semi-structured interviews	Content analysis
VanCauwenberg et al. (2012), [52]	Belgium	Adults > 65 years in urban or semi-urban areas	57	Walk-along interviews and structured interview	Content analysis
Zieff et al. (2012), [26]	USA	Residents > 18 years and city staff from low- and high-crime neighborhoods	101	Focus groups	Grounded theory
Casey et al. (2011), [41]	Australia	Men 25–65 years from low-SES neighborhoods	25	Semi-structured interview	Content analysis
Cassou et al. (2011), [50]	Brasil	Women > 60 years from low and high SES neighborhoods	25	Focus Group	Content analysis
Montemurro et al. (2011), [13]	Canada	Adults from urban city	63	Focus Group	Content analysis
Azar et al. (2010), [42]	Australia	Women 18–30 years, with and without depressive symptoms	40	Semi- structured interviews	Thematic analysis
Gallagher et al. (2010), [27]	USA	African American seniors >65 years in Detroit	21	Photovoice and Focus Groups	Content analysis
Grant et al. (2010), [36]	Canada	Adults > 65 years who resided in same neighborhood >2 years	75	Focus Groups	Not stated
Mathews et al. (2010), [28]	USA	Older adults > 50 years from different ethnic minority groups	396	Focus Groups	Thematic analysis
Annear et al. (2009), [48]	New Zealand	Older adults 65–91 years of high and low deprivation neighborhoods	63	Surveys and semi-structured interviews	Not stated
Caperchoine et al. (2009), [43]	Australia	Women belonging to women walking groups	78	Focus Groups	Thematic analysis
Burgoyne et al. (2008), [49]	Ireland	Adults from low-income neighborhoods	Not stated	Focus groups and unstructured interviews	Grounded theory
Dunn (2008), [29]	USA	African American women 45–65 years	14	Focus Groups	Content analysis
Strath et al. (2007), [30]	USA	Adults > 55 years from low and high walkable neighborhoods	37	Survey with semi-structured interviews.	Content analysis
Walker et al. (2007), [44]	Australia	Women 75–93 years living alone in the community	20	In-depth interviews	Grounded theory
Yen et al. (2007), [14]	USA	Women with at least one child < 18 aged 21–66 years in 3 neighborhoods	52	Focus groups	Thematic analysis
Ball et al. (2006), [45]	Australia	Women 18–65 years from high and low-SES neighborhoods	37	Semi-structured interviews	Thematic analysis
Lockett et al. (2005), [37]	Canada	Elderly 60–90 years from rural and urban neighborhoods	27	Photovoice and focus groups	Grounded theory
Burton et al. (2003), [46]	Australia	Adults 18–60 years from low, middle and high individual-level SES groups	60	Semi-structured interviews	Not stated
Eyler et al. (2002), [31]	USA	White, African-American, American-Indian and Latina women 20–50 years	Not stated	Focus Groups	Not stated
Eyler et al. (1998), [32]	USA	Women > 40 years	Not stated	Focus Groups	Not stated

SES: Socioeconomic status.

## Appendix B

**Table B1:** Summary of findings related specifically to the BE features and PA extracted from the reviewed studies (*n* = 36).

Author, Year of Publication, Reference	Functional	Safety	Aesthetics	Destination	Other Outcomes
Cleland et al. (2015), [12]	(+) Footpaths.	(-) Road safety related to large trucks and winding roads in rural. (-) Traffic density in urban areas.	(+) Nature changing with seasons in rural areas.	(-) Facility hours not meeting needs. (-) Not being able to walk/cycle places.	
Mitra et al. (2015), [33]	(-) Lack of benches, poor sidewalk quality.	(-) Absence of street lights.	(+) Nature and trees.	(+) Proximity to parks, access to shops.	
Mama et al. (2015), [22]		(-) Criminal activity.		(-) Lack of nearby PA (Physical activity) facility.	(-) Time, caretaking. (+) Social support.
Ivory et al. (2015), [47]			(+) Greenery was restorative. (+) Pleasantness/Beauty: running/walking in bush, beach as opposed to streets even if accessed by car.	(+) Open spaces (fields, playgrounds, cemeteries…). * Nearness of destinations appeared to be more influenced by easy accessibility by car or frequency of use.	(+) PA for social connection and mental restoration over and above specifically “health” reasons.).
Marquez et al. (2014), [23]		(-) Lack of safety: crime (gangs and drugs) especially after dark. (-) Racial tensions, lack of jobs, lack of social cohesion. (-) Non-crime safety (sidewalk cracks, traffic, weather, ice, snow, cold, extreme heat (fear of falling). (+) Longer time for elderly crossings, “elderly people present”.		(+) Elders walk to stores for PA and socialization.	(+) Family structure and passing on of PA values
Belon et al. (2014), [34]	(+) Direct public transit access to PA facilities. (+) Paths, sidewalks, shaded areas.	(-) Sidewalk cracks for seniors. (-) Surfacing materials under playgrounds. (+) Street lighting. (-) Traffic cyclist concerns. (-) Weather.	(+) Green spaces: peace. (+) Architecture. (-) Trash and vandalism, pollution (+) Promotion of group activities to counter crime.	(+) Recreation infrastructure (soccer fields, tennis courts) in walking distance for seniors and children, car owners reported unwilling to drive for recreation. (-) Parks that are not conveniently located. (-) Hours of operation of pools. (-) Expensive PA facilities.	(+) Access to information on local activities. (+) Social aspects: motivation (peer support) for PA. (-) Car culture and screen time. (+) Free PA programs.
Walton E. (2014), [24]	(+) Improved access in traffic areas through pedestrian bridges.	(+) Safety of parks may increase their use at all hours of the day.	(+) Parks and courtyards.	(+) Proximity to ethnic grocery store, proximity to parks increases their use. (+) Park as an area that creates social ties.	
Shuval (2013), [25]		(-) Neighborhood crime.		(-) Lack of exercise facilities and parks.	(+) Social pressure to walk.
Bellows-Riecken (2013), [35]	(-) Weather.		(-) Unaesthetic environments.		(+) Social involvement. (-) Conflicting with activities such as studies.
Eriksson et al. (2013), [51]		(-) Safety concerns more evident among females.	(+) Neighborhood greenness = well-being.	(+) Greenspaces, work, school, family, friends, leisure.	(+) “Hi factor”: inclusivity and joy from being greeted.
Kilgour et al. (2013), [38]		(-) Fear of dark (+) Familiarity of geography and people.			
Bjornsdottir et al. (2012), [53]	(+) Non-slippery sidewalks.	(-) Wind, ice, hills/stairs. (+) Familiar surroundings.	(+) Importance of “fresh air”.	(+) Good outdoor areas, proximity to shops.	(-) Low encouragement from family, staff and culture.
Lord et al. (2012), [40]		(-) Winter sleet, summer temperatures.		(+) Facility access.	(+) Positive community environment.
Mahmood et al. (2012), [54]	(+) Getting there: convenient public transit: both scheduling and infrastructure. (+) Comfort in movement: easily accessible sidewalks and walkways, safer parking lots, benches, drinking fountains (facilitator), clean public bathrooms. Shade, railings to stairs.	(-) Sidewalks ending, having to walk in the street, improper lighting. (-) Traffic hazards (cars speeding, heavy traffic, unsafe drivers), lack of crossings. (-) Signs of poverty, drugs, criminal activity, vandalism, poor housing.	(+) Beautiful scenery, rivers, trees, mountains, flowers and sculptures.	(+) Diversity of Destinations: parks, greenspace, markets, festivals.	(+) community based programs. (+) peer support: family, community gardening and activities, intergenerational programs.
Stathi et al. (2012), [39]	(+) Seating along walking routes, wide pavements, good bus service. (-) Hills.	(-) Weather and darkness. (-) Fear of crime, lack of police presence.	(+) Attractive local environments. (-) Poor maintenance gardens. Lack of cleanliness, traffic noise, cars parked on sidewalks.	(+) Local amenities in walking distance (high impact) such as post office, newsagent, food stores, shops, PA facilities. (+) Accessible green space.	(+) Friendly neighbors. (+) Encouragement to be active, exercise companion. (+) Past active habits.
VanCauwenberg et al. (2012), [52]	(+) Walking facilities: sidewalks quality, crossings, benches (+) Connectivity (-) Increased risk of falling.	(-) Traffic safety (bus, behavior of road users including cyclists on sidewalk and careless car drivers). (-) Safety from crime. (-) Weather.	(+) Buildings, natural elements. (-) Noise, smell.	(+) Access to facilities. (+) Non-residential uses of land. (+) Public transit.	(+) Familiarity, social contact.
Zieff et al. (2012), [26]		(-) Violent Crime and non-violent crime (litter, garbage, dog waste, drug paraphernalia). (-) Low SES neighborhoods: vacuums where lack of function led to low police involvement/crime.		(+) Parks, community gardening, YMCA. (+) Increased access to information on places to go to get PA was important.	(-) Racial profiling in some low income neighborhoods.
Casey et al. (2011), [41]		(-) Fear of violent neighborhoods preventing men from leaving house: stems from lack of community trust.		(-) PA facilities lacking quality (no people to explain machines).	(+) Affordability (few inactive men recognized potential for free PA).
Cassou et al. (2011), [50]		(-) Safety was more of a concern ore among low SES. (-) Fear of injury.			(-) Lack of social support among high SES women. (-) Environmental barriers amongst low SES women.
Montemurro et al. (2011), [13]	(-) Lack of path connectivity and quality.	(-) Winter walking and traffic.	(+) Natural walking area such as river in walking distance promoted walking for leisure.	(-) Adults preferred car use to travel and report walking mainly for leisure. (+) Dog park important. (+) Recreational infrastructure for children. (-) Matching infrastructure to community needs (unused baseball diamonds).	(-) Cost barrier for PA. (+) Information on local recreational activities desired. (+) Social capital and community.
Azar et al. (2010), [42]	(+) Footpaths.	(+) Proper lighting.		(+) Facilities (tennis, courts), dog park. (+) Community center activities that are publicized.	(-) Women with depressive disorder more likely to report past negative PA experiences.
Gallagher et al. (2010), [27]	(+) Shade, shoveled sidewalks. (-) Sidewalks that end, ice. (+) Walking trails. (+) Amenities: places to eat, use washroom and rest.	(-) Criminal activity. (-) Isolated trails with poor visibility. (-) Fear of dogs. (+) Senior patrol, police, early morning walking.	(+) Peaceful, beautiful scenery. (+) Gardens and parks. (-) Vacant houses, overgrown lots, trash. (+) Presence of animals: brids and squirrels. (+) Enjoyment of fresh air.	(+) Historical destinations with meaning.	(+) Presence of people and familiar faces. (+) Seeing others being physically active.
Grant et al. (2010), [36]	(+) Getting around: fitting walking within an integrated transportation system that includes the elderly. (-) Experiencing ambiguity (right of way).	(-) Navigating hostile walking environments such as ending sidewalks.	(+) Personal meanings given to green space.		(+) Meaningful relationships.
Mathews et al. (2010), [28]		(-) Fear of falling.		(-) Distance to recreation facilities as a barrier. (+/-): Church as barrier or support.	(-) Assimilation for indigenous people. (+) Having non-PA related transportation be less convenient to encourage walking.
Annear et al. (2009), [48]		(-) Traffic/speed/noise/air pollution.	(+) Attractive and walkable surroundings (parks, gardens, attractive paths). (+) Pride in ownership.	(+) Well-served leisure environment.	(+) Social support.
Caperchoine et al. (2009), [43]		(-) Traffic/lighting/animals. (-) Gangs/unfamiliar people.			(-) Childcare. (-) Lack of support.
Burgoyne et al. (2008), [49]		(-) Lack of cleanliness, garbage (cans, glass, old fridges and cars…). (-) lack of lighting, gangs.		(+) Need for gym/pools in walking distance for families with only 1 car who rely on public transit.	(+) Community contentment: personally, socially and environmentally content.
Dunn (2008), [29]		(-) Unsafe neighborhood.			(-) Lack of family support, family obligations. (+) Most compelling reason to walk in was to help others.
Strath et al. (2007), [30]	(+) Presence and maintenance of sidewalks. (+) Traffic control for streets, bicycle lanes and trails.	(+) Separating walkers and cyclists from motorized traffic. (+) Sense of personal safety for women especially.	(+) Living things like trees. (+) Scenic route chosen even if it is longer.	(+) Retail and entertainment (restaurants), parks, recreation, natural areas, libraries, churches. (+) Older adults willing to walk for transportation 20 to 45 min to engage in pleasurable and purposeful activities.	(+) Social environments and social support.
Walker et al. (2007), [44]	(-) Elderly felt that lack of ramp access excluded them from certain areas.	(-) Elderly women took inordinate steps to ensure that door and windows were locked and secure.	(+) Outdoors areas with birds counteracted feelings of loneliness.	(+) Access to destinations within walking distance was comforting for elderly who were going to stop driving soon.	(+) Social capital. (-) Difficulty to make new neighbor friends in elderly.
Yen et al. (2007), [14]		(-) Unsafe parks in low SES. (+) Improved maintenance and police presence. (-) Gangs, drug dealing, loitering, explicit sexual behavior.		(-) Fast food destinations result in car traffic and unsightly garbage. (-) Low-SES women perceived neighborhood area is 1/4th that of high-SES women. (+) Parks/facilities in high-SES areas.	(-) Neighborhood characteristics vary by income and inform adults’ opinions of hazards and resources as well as their behaviors.
Ball et al. (2006), [45]		(-) Crime issue in low SES groups.		(+) Low SES more likely to participate in transport related PA (walking cycling) as opposed to high SES (gym, sports…).	(+) Family PA in low-SES. (+) Dog walking common to all SES groups.
Lockett et al. (2005), [37]	(+) Amenities such as benches and washrooms.	(-) Traffic hazards (crosswalks, light timing, cars). (-) Fall hazards: slopes, ending sidewalks, cracks, lack of railings, stairs, snow, ice.	(+) Waterfalls and trees mentioned.		
Burton et al. (2003), [46]		(+) Environmental safety		(+) Aesthetics.	(+) Social support.
Eyler et al. (2002), [31]	(-) Lack of sidewalks and uneven pavement for walking.	Weather and daylight. (-) Traffic. (-) Presence of homeless, drug dealers or drive-by shootings. (-) Dust, traffic, insects, dogs.		(-) Distance to facilities makes walking difficult.	(-) Multiple women’s cultural duties/roles: wife, daughter, mother, worker. (+) Social support. (+) Traditional/cultural PA.
Eyler et al. (1998), [32]		(-) Fear of darkness outdoors and crime.	(+) Scenic places to exercise.		(+) Dancing (powow, …). (-) lack of social network.

(+): Supports for PA, (-): barriers for PA, *: factors influencing perceived supports and barriers, SES: socioeconomic status.
